# Annotations

**Published:** 1906-08-25

**Authors:** 


### ANNOTATIONS.

### Examiners and Experts.

The question has recently been raised whether into the final examinations for medical qualifications examiners having special knowledge in particular departments should be introduced. In the earlier subjects of the medical curriculum the necessity for expert examiners has long been recognised, and practising physicians and surgeons now rarely appear as examiners in chemistry, biology, and physics, or even in physiology and anatomy. Public health and forensic medicine among the later subjects are also usually placed in the hands of those who have special and expert knowledge in these departments. Hence at first sight it seems not unreasonable to add to the examining staff of the final examinations those who have made a special study of the manifestations of disease in such organs as the eye, ear, throat, and skin. Objection has been raised that such examiners will be apt to press their particular departments beyond what is reasonable, and to demand a standard and degree of knowledge beyond what in the circumstances can fairly be expected. This, we think, is an argument of slight weight, provided only that the examiners have had sufficient experience as teachers of students. It is on this point that there should be an essential demand made of those who aspire to conduct examinations. The more expert and equipped with knowledge the examiner the better, for no one is so dangerous to the candidate as he who is himself but half informed and is able therefore to find accuracy only in the answer he expects. It may be granted that the expert who has not been disciplined and chastened by prolonged experience as a teacher would probably prove a very incompetent and absurd examiner. But if such experience has been enjoyed, indulgence in absurd demands is hardly likely to appear. Whether the change suggested in the final examination in medicine is or is not necessary may be open to question, but if it is adopted there need be no fear of the consequences so long as the examiners are experienced teachers of medical students.

### Patent Medicines.

The love of physic which characterises certain sections of the community has been the text for many homilies, but these, as is proverbially the case with such exhortations, cannot boast any large measure of success. Upon this popular appetite is founded the widespread use of the so-called "patent" medicines, the extravagant pretensions of which appear to be an increasing quantity. Now and again some legal process discovers the true nature of these pretensions or shows the enormous profits which they yield to those who advance them. By some the Government stamp is believed to act as a decoy to the public, and certainly it is a sorry thing that the national exchequer should receive a share of what in many cases are essentially fraudulent gains. Recently, both in the medical and lay press, there have been published analyses of a number of these much vaunted remedies, and the proclamation of their composition and original cost may do something to secure public enlightenment. An occasional intimation cannot, however, successfully oppose organised repetition and advertisement. What is wanted is a compulsory announcement of the true nature of the contents upon every package of these secret preparations, for which at present, under artificial and fancy names, the public pay extravagant prices.

### The Prevention of Malaria.

Recent letters in the *Times* by Professor Ross and Dr. Hamilton Wright call attention to the very successful results of two anti-malarial campaigns. One of these is the well-known Ismailia experiment, the other the Port Swettenham. The importance of the latter, as viewed by its results, is by no means the less on this account. Initiated by Dr. Hamilton Wright, when Director of the Institute for Medical Research in the Federated Malay States, the work has subsequently been carried on and supplemented by two of the local Government medical officers—Drs. Travers and Watson. After having been in existence now for some years, the latter report an extraordinary diminution in the number of malarial cases in the treated area and a consequently greatly reduced death-rate. A quotation from Dr. Wright's letter in the *Times* will indicate how this has been achieved. After a severe outbreak of malaria "a Commission was at once appointed, composed of medical men, railway and dock officials, and instructed to devise measures for the suppression of malaria and to otherwise sanitise the port. The recommendations of the Commission involved an outlay of from £10,000 to £12,000, a vast sum one would say to protect the lives of a few officials and their underlings." "The Government without hesitation accepted the recommendations of the Commission, and the new port was dyked, drained, levelled, and cleared. Since these sanitary measures were initiated there has been scarcely a case of malaria at the port, and from being an unhealthy, shunned swamp, the port is now sought by officials as a desirable billet." Such striking examples are very gratifying, and should stimulate other Colonies to further efforts to stamp out the disease of all others that has made the tropics so unhealthy.

### A French Mission to Brazzaville.

France, following the example of other countries, now proposes to send out a mission to Africa for the study of sleeping sickness and other tropical diseases. The mission—which will consist of Major Martin, who has worked at Saigon and at Lille in the Pasteur Institutes; Dr. Lebeuf; M. Roubaud, of the Museum; and M. Weiss, a young naturalist who has spent some years in Tunis—will leave Paris in October for Brazzaville and establish a permanent central laboratory before commencing the study of the disease up country. The main object of the expedition will be to fight the tsetse fly, and as the general points of the disease are now so well worked out, this is the correct line to adopt. It is the prophylaxis of the disease that must now be followed; the old adage "Prevention is better than cure" specially appeals to this dreaded disease.

